# Trajectory of Urine Parameters by Adding Herbal Kampo Medicine Goreisan to Tolvaptan in Patients with Congestive Heart Failure

**DOI:** 10.3390/jcm13247523

**Published:** 2024-12-11

**Authors:** Kota Kakeshita, Teruhiko Imamura, Yuki Hida, Hiroshi Onoda, Tsutomu Koike, Koichiro Kinugawa

**Affiliations:** The Second Department of Internal Medicine, University of Toyama, Toyama 930-0194, Japan

**Keywords:** diuretics, natriuresis, aquaresis, fractional excretion, oryeongsan, wulingsan

## Abstract

**Background:** Even in current guideline-directed medical therapy, including recently introduced vasopressin type 2 receptor antagonist tolvaptan, congestion has not been resolved in patients with heart failure. Kampo medicine goreisan has been receiving considerable attention as an additional therapy for patients who are refractory to conventional diuretics therapy, including tolvaptan. However, the impact of goreisan on urine electrolytes remains uncertain. **Methods:** Patients with congestive heart failure who received goreisan as an add-on therapy to tolvaptan-incorporated medical therapy were prospectively included. The changes in urine parameters during the first 24 h were assessed as a primary concern. Baseline factors associated with an increase in urine sodium excretion were investigated. **Results:** A total of 21 patients were included. The median age was 81 (77, 86), and 13 (62%) were men. Twenty-four hours after the initiation of goreisan, urine osmolality decreased significantly, urine sodium level remained unchanged, urine potassium and glucose levels decreased significantly, urine urea nitrogen level tended to decrease, and urine volume tended to increase. The fractional excretion of sodium tended to increase. Baseline plasma B-type natriuretic peptide level had a positive correlation with a change in fractional excretion of sodium from baseline to day 1 (r = 0.52, *p* = 0.015). **Conclusions:** Goreisan may increase urine volume via aquaretic and natriuretic effects in patients with congestive heart failure receiving tolvaptan-incorporated medical therapy. Goreisan may have the ability to “modulate” fluid balance depending on congestion status.

## 1. Introduction

Guideline-directed medical therapy, which includes beta-blockers, renin–angiotensin–aldosterone system inhibitors, and sodium-glucose cotransporter 2 inhibitors, together with appropriate diuretics, has been shown to reduce mortality and morbidity in patients with chronic heart failure, particularly those with reduced left ventricular ejection fraction [1,2]. However, congestion remains an unsolved issue against these medications [3].

Conventional loop diuretics are pivotal in managing congestion, but they come with several limitations, including stimulation of renin-angiotensin system, induction of hyponatremia and intravascular hypovolemia, related hypotension, and deterioration of renal function [4]. Moreover, some patients exhibit resistance to conventional diuretics, especially those with advanced renal failure who require substantial doses of these agents.

For patients who have an inadequate response to conventional diuretics, particularly in the context of advanced renal dysfunction, the vasopressin type 2 receptor antagonist tolvaptan has been considered [5,6]. Tolvaptan inhibits aquaporin-2-mediated reabsorption of free water in the renal collecting ducts, thereby promoting its excretion in the urine and leading to decreased urine osmolality and incremental urine volume. Nonetheless, some patients remain refractory to tolvaptan-based therapy, particularly those with impaired collecting duct function [7].

In recent years, herbal Kampo medicine has attracted great attention as an additional medical treatment, particularly in patients refractory to conventional Western medications [8,9]. Among these traditional medicines, goreisan (Tsumura & Co., Tokyo, Japan) is one of the most established herbal formulations, composed predominantly of five herbs: Alisma Tuber, Poria Sclerotium, Atractylodes Lancea Rhizome, Cinnamon Bark, and Polyporus Sclerotium. Goreisan is widely prescribed for conditions characterized by disrupted body water balance [10,11]. Importantly, goreisan 7.5 g/day is reimbursed in Japan for edematous diseases, nephrotic syndrome, or uremia, accompanying thirst and oliguria.

Recent experimental and clinical studies have highlighted the potential of goreisan to alleviate congestion that is refractory to conventional diuretics by suppressing aquaporin-2-mediated reabsorption of free water, particularly in patients with residual systemic congestion [12]. However, the detailed biological impact of goreisan on urine electrolytes remains poorly understood. Elucidating these effects would enhance our understanding of goreisan’s efficacy in regulating body fluid balance and inform the development of therapeutic strategies incorporating goreisan for patients with congestive heart failure.

In this study, we examined the short-term effects of goreisan on urine electrolyte composition in patients with congestive heart failure who were already receiving tolvaptan-based medical therapy.

## 2. Materials and Methods

### 2.1. Patient Selection

This study included patients who were hospitalized for congestive heart failure and exhibited congestion that was refractory to tolvaptan-based medical therapy, consisting of loop diuretics, tolvaptan, and guideline-directed heart failure medications. These patients received goreisan as an adjunctive treatment between April 2022 and July 2024. Patients who received mechanical circulatory support or underwent percutaneous or surgical interventions during the observation period were excluded. Additionally, patients who were dependent on hemodialysis did not receive goreisan and were, therefore, not included in this study.

### 2.2. Study Design

This was a prospective, single-center, observational study involving patients who received goreisan as an add-on therapy to tolvaptan-based medical treatment. The spray-dried extract powder of goreisan (Tsumura & Co., Tokyo, Japan) was administered at a dosage of 7.5 g per day, divided into three doses per day, and continued for a minimum of seven days in all participants. Urine parameters were measured at baseline and again on day 1 following the start of goreisan administration. The primary objective was to evaluate the trend in urine parameters within the first 24 h after starting goreisan therapy. Written informed consent was obtained from all participants, and the study protocol was approved by the institutional review board.

### 2.3. Clinical Data Collection

Baseline characteristics, including demographics, comorbidities, laboratory values, echocardiographic findings, and medications just before the administration of goreisan, were systematically collected. Blood and urine samples were obtained from all patients at fasting condition. Urine biomarkers were assessed at baseline and day 1, including urine osmolality, sodium, potassium, glucose, and urea nitrogen. The fractional excretions of each filtered parameter were calculated by the following formulas: for example, the fractional excretion of sodium (%) = sodium clearance/creatinine clearance × 100 = (urine sodium [mEq/L]/serum sodium [mEq/L])/(urine creatinine [mg/dL]/serum creatinine [mg/dL]) × 100. In patients where 24 h urine collection was possible, free water clearance was calculated using the following formulas: free water clearance (mL/min) = urine volume (mL/min) − (urine osmolality [mOsm/kg H_2_O] × urine volume [mL/min])/serum osmolality (mOsm/kg H_2_O). In total, 24 h urine volumes were measured at baseline and day 1 after the initiation of goreisan administration.

### 2.4. Statistical Analysis

Continuous variables were reported as medians with interquartile ranges (25th and 75th percentiles), while categorical variables were presented as counts and corresponding percentages. The trend in clinical parameters between the two time points was evaluated using the Wilcoxon signed-rank test for continuous variables. To assess the change in natriuresis following the administration of goreisan, the difference in the fractional excretion of sodium between baseline and day 1 was calculated as a primary concern. Baseline variables potentially associated with changes in the fractional excretion of sodium after the initiation of goreisan were analyzed using linear regression analysis.

Significance was defined as a two-tailed *p*-value less than 0.05. The statistical analyses were performed using SPSS Statistics 23 (SPSS Inc., Armonk, IL, USA).

## 3. Results

### 3.1. Baseline Characteristics

A total of 21 patients were included (Table 1). All goreisan therapy was initiated during the index hospitalization. The median age was 81 (77, 86) years, and 13 (62%) were men. All patients had received loop diuretics and tolvaptan and had been hospitalized for congestive heart failure. Left ventricular ejection fraction was 53% (46%, 66%), and two (10%) patients were <40% of left ventricular ejection fraction, six (29%) were 40–49%, and thirteen (62%) were ≥50%. The plasma B-type natriuretic peptide level was 372.9 (137.7, 513.0) pg/mL, and the estimated glomerular filtration rate was 32.9 (17.7, 44.9) mL/min/1.73 m^2^. Goreisan was continued without any drug-related adverse events, except for one patient who terminated goreisan due to suspected hepatic injury.

### 3.2. Changes in Urine Parameters During the First 24 h

The trends of urine parameters during the first 24 h after the initiation of goreisan were displayed in Table 2. Urine osmolality decreased significantly from 378 (304, 485) to 351 (273, 410) mOsm/kg H_2_O. In the urine concentration of parameters that make up urine osmolality, i.e., urine sodium, potassium, glucose, and urea nitrogen, urine sodium levels remained unchanged (from 45 [40, 70] to 44 [33, 71] mEq/L), whereas urine potassium and glucose levels decreased significantly (from 24 [16, 33] to 21 [13, 28] mEq/L, form 57 [5, 742] to 24 [3, 332] mg/dL, respectively). Urine aquaporin-2 levels tended to decrease (from 0.97 [0.55, 3.01] to 0.76 [0.31, 2.92] ng/mL). Urine volume tended to increase (from 1089 [807, 1455] to 1358 [931, 1613] mL/day).

Such trends were almost similar regardless of the concomitant administration of sodium-glucose cotransporter 2 inhibitors (Table 2).

### 3.3. Changes in Fractional Excretions During the First 24 h

**Figure 1:**
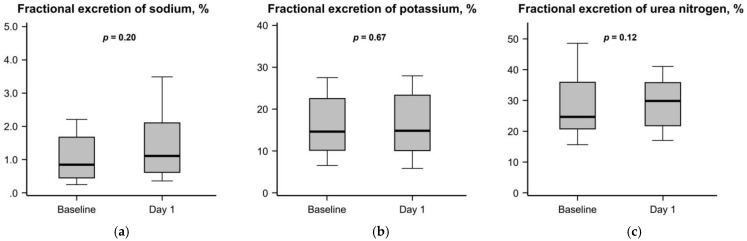
Change in fractional excretions of each filtered parameter between baseline and one day after the initiation of (**a**) goreisan: sodium; (**b**) potassium; and (**c**) urea nitrogen. Variables were compared between the two groups by using the Wilcoxon signed-rank test. Fractional excretion of sodium and urea nitrogen tended to increase following the initiation of goreisan, whereas those of potassium remained statistically unchanged.

The trend in fractional excretion during the first 24 h after the initiation of goreisan was displayed in Figure 1a–c. The fractional excretion of sodium tended to increase (from 0.77% [0.49%, 1.52%] to 1.11% [0.62%, 2.10%]) (Figure 1a). The fractional excretion of potassium remained unchanged (from 15.1% [10.2%, 22.2%] to 14.9% [10.3%, 23.2%]) (Figure 1b). The fractional excretion of urea nitrogen tended to increase (from 25.2% [20.7%, 34.9%] to 29.8% [21.7%, 35.8%]) (Figure 1c).

### 3.4. Changes in Free Water Clearance During the First 24 h

**Figure 2:**
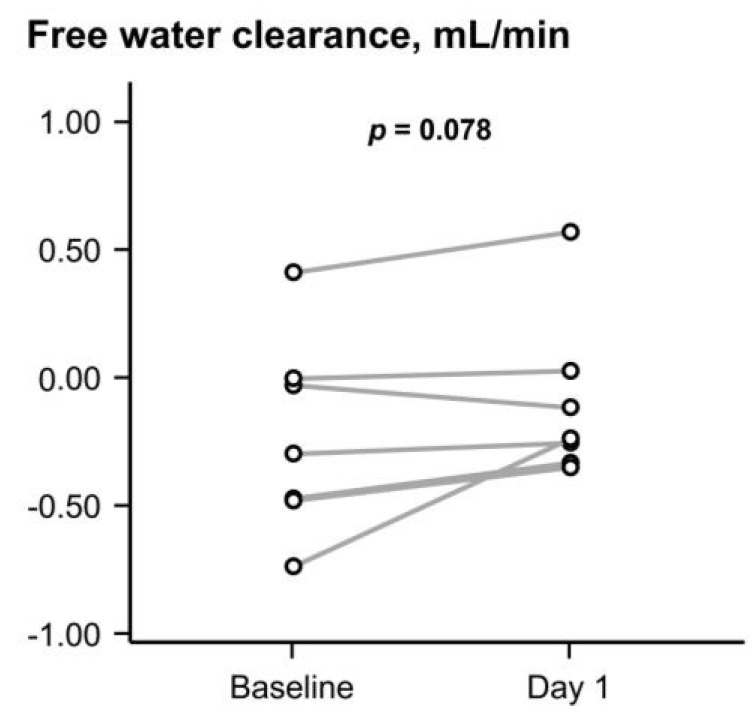
Change in free water clearance. Variables were compared between the two groups by the Wilcoxon signed-rank test. Free water clearance tended to increase following the initiation of goreisan.

Seven (33%) patients had 24 h urine collection. In these patients, the free water clearance tended to increase (*p* = 0.078; Figure 2).

### 3.5. Baseline Plasma B-Type Natriuretic Peptide Level and Fractional Excretion of Sodium

**Figure 3:**
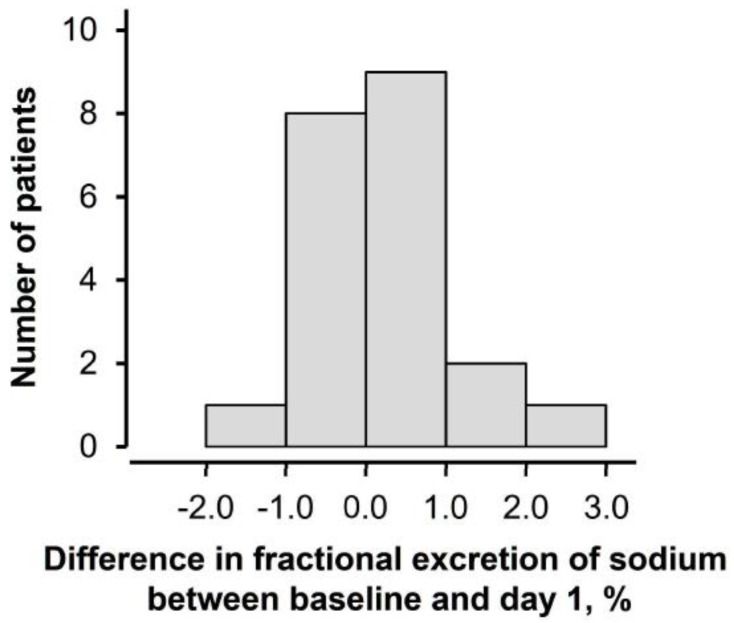
Distribution of change in fractional excretion of sodium.

**Figure 4:**
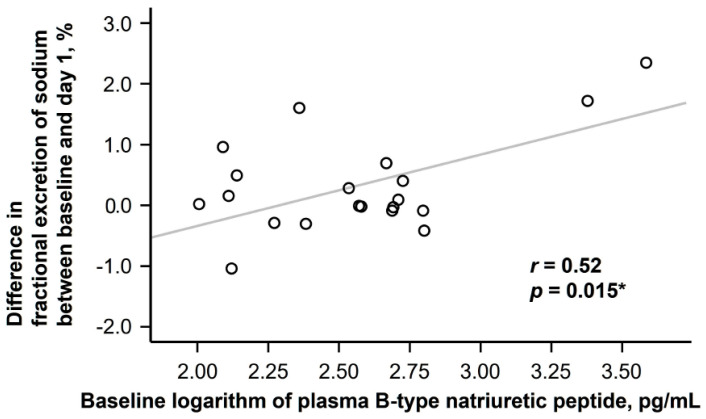
Correlation between baseline plasma B-type natriuretic peptide and change in fractional excretion of sodium. The correlation was assessed using Pearson’s correlation coefficient. * *p* < 0.05. Patients with higher baseline plasma B-type natriuretic peptide levels, indicating the presence of congestion, tended to encounter greater increase in fractional excretion of sodium during 24 h goreisan therapy.

The difference in fractional excretion of sodium between baseline and day 1 distributed widely ranging between −1.04% and 2.35% (Figure 3). The baseline logarithm of plasma B-type natriuretic peptide level had a moderate correlation with the change (r = 0.52, *p* = 0.015; Figure 4). According to the findings of univariable and multivariable linear regression analyses, baseline plasma B-type natriuretic peptide level was independently associated with the change in fractional excretion of sodium among potential clinical parameters (Table 3).

## 4. Discussion

In this prospective study, we specifically evaluated the effects of goreisan on urine electrolyte parameters in patients with congestive heart failure who were receiving tolvaptan-based medical treatment (i.e., tolvaptan, loop diuretics, and heart failure medications). The principal findings are as follows: (1) Urine volume increased, and urine osmolality decreased, indicating that goreisan exerts an aquaretic effect. This effect persisted until the following morning when goreisan was administered at the standard dosage (7.5 g/day, three times per day). Correspondingly, there was a trend toward increased free water clearance. (2) Among the urine electrolyte concentration parameters contributing to urine osmolality, the sodium concentration remained unchanged, while the concentrations of other components, such as potassium, glucose, and urea nitrogen, decreased. This suggests that sodium excretion in the urine increased after the initiation of goreisan therapy. Consistent with this observation, the fractional excretion of sodium also tended to increase, highlighting the natriuretic effect of goreisan, in addition to its aquaretic effect. Notably, baseline plasma B-type natriuretic peptide levels, indicating the presence of congestion and volume overload, were independently associated with an increase in the fractional excretion of sodium during the one-day goreisan therapy.

### 4.1. Impact of Goreisan on Aquaresis

Several previous experimental studies have highlighted the potential of goreisan to modulate aquaporin-mediated signaling cascades, thereby contributing to the regulation of body fluid balance. In 5/6 nephrectomy rat models (a model of chronic kidney disease), the administration of goreisan was shown to decrease aquaporin-2 expression in the kidneys [13]. In another experimental model involving saline-loaded rats (a heart failure model), goreisan administration did not alter the expression of the vasopressin type 2 receptor but did reduce the expression of aquaporin-2 and -3 [14]. Furthermore, in a murine inner medullary collecting duct cell line, goreisan administration suppressed the expression of aquaporin-2 through the inhibition of the cyclic AMP signaling cascade [15].

In the recent clinical literature, both urine cyclic AMP and urine aquaporin-2 levels decreased following goreisan administration in patients with congestive heart failure [12]. Our findings demonstrated that free water clearance tended to increase after the initiation of goreisan, which supports these previous findings. Goreisan probably exerts its aquaretic effect by directly regulating aquaporin-2 and -3, while tolvaptan exerts its aquaretic effect by antagonizing the vasopressin type 2 receptor. The therapeutic mechanisms of goreisan appear to differ from those of tolvaptan, resulting in a synergistic effect that enhances free water excretion in the urine. Although further studies involving serial sampling are warranted, the impact of goreisan may persist overnight, potentially preventing hemodynamic deterioration that can result from the acute diuretic response usually associated with conventional loop diuretics.

### 4.2. Impact of Goreisan on Natriuresis

In an experimental study involving spontaneously hypertensive rats, goreisan administration was shown to increase urine volume and urinary sodium excretion, which was subsequently followed by a reduction in blood pressure. In the renal cortex of these rats, the expression of sodium-hydrogen exchanger 3 was decreased, the expression of the angiotensin I converting enzyme-angiotensin II type 1 receptor was suppressed, and the expression of the angiotensin II type 2 receptor/angiotensin I converting enzyme 2-Mas receptor was enhanced. The authors concluded that goreisan may improve sodium and water retention by modulating the renin-angiotensin and natriuretic peptide systems [16,17,18]. In another experimental study using hypertensive rats, goreisan administration reduced blood pressure by suppressing the renin–angiotensin–aldosterone system and prevented the progression of renal fibrosis [19].

The present study supports these findings by demonstrating an increase in the fractional excretion of sodium during goreisan therapy for the first time in real-world clinical practice. Although the detailed underlying mechanisms remain unclear, unlike conventional loop diuretics, goreisan may exert its natriuretic effect via suppressing renin-angiotensin system. Such an impact of goreisan may have a favorable prognostic impact on heart failure patients by suppressing the neuro-hormonal system. Furthermore, enhancing natriuresis is favorable for treating patients with congestive heart failure, in whom extra-cellular volume is increased.

Moreover, the degree of natriuresis was correlated with baseline plasma B-type natriuretic peptide levels, which indicates the presence of congestion and volume overload [20]. This suggests that goreisan may have the ability to “modulate” fluid balance according to the body’s status rather than simply “forcing” diuresis. For instance, goreisan may increase urine volume in heart failure patients with huge congestion, whereas the urine volume may rather be suppressed by goreisan administration in patients with relative hypovolemia. This distinctive characteristic of goreisan may offer advantages over conventional diuretics by reducing the risk of hemodynamic deterioration and undesired hypotension. Notably, accurate assessment of congestion is often challenging in the elderly cohorts. The long-term prognostic impact of goreisan therapy remains an area of future investigation.

### 4.3. Future Concerns

We have several concerns about the findings of the present study. First, while the study demonstrates the aquaretic and natriuretic effects of goreisan, further research is necessary to fully understand the therapeutic mechanisms, particularly its regulation of aquaporin pathways and its synergistic interactions with tolvaptan. This includes conducting additional serial sampling studies to assess the persistence and clinical implications of these effects.

Another significant concern is the long-term impact of goreisan therapy on patient outcomes in heart failure management. While short-term benefits are observed, the potential for improved prognosis or the mitigation of complications like hemodynamic deterioration remains to be established through further investigation.

We administered 7.5 g/day of goreisan. The next concerns are multiple dosing comparisons and drug interaction studies under multiple clinical data monitoring.

The precise mechanisms underlying goreisan’s natriuretic effects also require clarification, particularly its apparent modulation of the renin-angiotensin and neurohormonal systems, which differ from those of conventional diuretics. Understanding these pathways could provide insights into its unique ability to modulate fluid balance according to the patient’s physiological status, such as enhancing urine output in cases of congestion while preventing excessive diuresis in hypovolemic conditions.

Moreover, the application of goreisan in specific populations, such as elderly patients, where assessing congestion is challenging, presents another area for exploration. The study highlights the importance of refining strategies to incorporate goreisan into treatment regimens without causing adverse effects like hypotension or hemodynamic instability.

Lastly, broader implications for goreisan’s role in heart failure therapy need to be addressed, particularly in comparison to or combination with traditional diuretics. Future studies are essential to optimize its use and evaluate its potential for enhancing long-term outcomes while minimizing risks.

### 4.4. Limitations

This study focused on the use of goreisan therapy in individuals who were refractory to tolvaptan-based medical treatment, resulting in a small sample size. In the current clinical landscape, it may be challenging to initiate goreisan therapy before tolvaptan, given the established evidence supporting the latter. Similarly, it is ethically challenging to treat patients with congestive heart failure by goreisan alone, given the established evidence of loop diuretics and tolvaptan. This study serves as a proof-of-concept, and further studies with larger sample sizes are needed to validate our findings. It is possible that several statistical outcomes could have reached significance with an increased sample size. Standardized medication protocol, diet monitoring, and sophisticated statistical methodologies, including robust multivariable analyses, sensitivity testing, and effect size calculation, are warranted in the next studies. Notably, we need randomized control trials to compare two groups: gorensan + tolvaptan arm versus tolvaptan alone to establish robust evidence of goreisan add-on therapy.

Owing to the observational nature of this clinical study, the potential influence of other medications cannot be entirely excluded. In real-world clinical practice, it is difficult to administer goreisan as monotherapy in patients with congestive heart failure. However, by the next morning, the effects of conventional diuretics are expected to have diminished. Notably, urine electrolytes exhibited significant changes even within one day following the initiation of goreisan therapy. Further research is required to explore the long-term impact of goreisan on urine parameters.

## 5. Conclusions

Goreisan may enhance urine volume through both aquaretic and natriuretic effects in patients with congestive heart failure, thereby preventing intra-vascular hypovolemia and improving congestion. Diuretic resistance is one of the challenges for clinicians. Goreisan may be a promising tool to treat patients who are refractory to conventional diuretics therapy, including tolvaptan. Furthermore, goreisan may be a “modulator” of fluid balance, not simply forcing diuresis. It is particularly promising for elderly patients, because their fluid balancing point is narrow, and an accurate assessment of fluid balance is challenging in this cohort.

The observed short-term benefits suggest that goreisan could offer a complementary approach to current diuretic treatments. Future research should focus on evaluating the long-term impact of goreisan on urine electrolytes and overall fluid balance. Additionally, studies are needed to explore the potential benefits of integrating goreisan into therapeutic strategies for managing congestive heart failure, particularly in patients who are refractory to conventional diuretics. Such investigations could help optimize treatment protocols and improve patient outcomes in this challenging clinical context.

## Figures and Tables

**Table 1:** Baseline characteristics.

Variables	*n* = 21
Demographics	
Age, years	81 (77, 86)
Men	13 (62%)
Body mass index, kg/m^2^	21.9 (20.2, 23.6)
Systolic blood pressure, mmHg	107 (95, 127)
Pulse rate, beat per minutes	71 (66, 83)
Comorbidity	
Coronary artery disease	9 (43%)
Atrial fibrillation	8 (38%)
Diabetes mellitus	13 (62%)
Laboratory data	
Hemoglobin, g/dL	9.8 (9.2, 13.0)
Serum albumin, g/dL	2.9 (2.7, 3.2)
Serum sodium, mEq/L	140 (138, 143)
Serum potassium, mEq/L	4.2 (3.8, 4.7)
Blood urea nitrogen, mg/dL	40.6 (25.4, 55.6)
Estimated GFR, mL/min/1.73 m^2^	32.9 (17.7, 44.9)
Logarithm of plasma BNP, pg/mL	2.6 (2.1, 2.7)
Plasma arginine vasopressin concentration, pg/mL	3.1 (2.4, 5.2)
Plasma renin activity, ng/mL/h	9.4 (1.2, 13.9)
Plasma aldosterone concentration, pg/mL	13.7 (4.0, 46.7)
Urine protein, g/g of creatinine	0.24 (0.15, 0.41)
Echocardiography findings	
Left atrial diameter, mm	48 (40, 51)
Left ventricular end-diastolic diameter, mm	52 (42, 58)
Left ventricular ejection fraction, %	53 (46, 66)
Moderate or greater mitral regurgitation	3 (14%)
Moderate or greater tricuspid regurgitation	7 (33%)
Medications	
Dose of tolvaptan, mg/day	7.5 (3.75, 15)
Equivalent dose of furosemide, mg/day	20 (20, 40)
ACE inhibitor or ARB	6 (29%)
Angiotensin receptor-neprilysin inhibitor	14 (67%)
Mineralocorticoid receptor antagonist	16 (76%)
Sodium-glucose cotransporter 2 inhibitor	11 (52%)
Beta-adrenergic blocker	17 (81%)

Continuous variables are presented as median and interquartile. Categorical variables are presented as numbers and percentages. GFR, glomerular filtration rate; BNP, B-type natriuretic peptide; ACE, angiotensin-converting enzyme; ARB, angiotensin II receptor blocker.

**Table 2:** Changes in urine osmolality and its breakdown during the first 24 h after the initiation of goreisen.

Variables	Baseline	Day 1	*p*-Value
Total (n = 21)			
Urine osmolality, mOsm/kg H_2_O	378 (304, 485)	351 (273, 410)	0.0057 *
Urine sodium, mEq/L	45 (40, 70)	44 (33, 71)	0.87
Urine potassium, mEq/L	24 (16, 33)	21 (13, 28)	0.040 *
Urine glucose, mg/dL	57 (5, 742)	24 (3, 332)	0.026 *
Urine urea nitrogen, mg/dL	431 (249, 582)	348 (301, 488)	0.23
Urine aquaporin-2, ng/mL	0.97 (0.55, 3.01)	0.76 (0.31, 2.92)	0.083
Urine volume, mL/day	1089 (807, 1455)	1358 (931, 1613)	0.38
With sodium-glucose cotransporter 2 inhibitor (n = 11)			
Urine osmolality, mOsm/kg H_2_O	395 (320, 539)	355 (249, 410)	0.042 *
Urine sodium, mEq/L	45 (40, 69)	44 (34, 69)	0.25
Urine potassium, mEq/L	28 (19, 35)	20 (13, 30)	0.066
Urine glucose, mg/dL	712 (65, 1350)	284 (45, 1177)	0.083
Urine urea nitrogen, mg/dL	490 (264, 578)	348 (286, 502)	0.24
Urine aquaporin-2, ng/mL	0.98 (0.62, 6.55)	0.86 (0.44, 3.18)	0.10
Urine volume, mL/day	1248 (901, 1562)	1358 (1190, 1700)	0.70
Without sodium-glucose cotransporter 2 inhibitor (n = 10)			
Urine osmolality, mOsm/kg H_2_O	354 (307, 458)	326 (289, 438)	0.084
Urine sodium, mEq/L	55 (34, 70)	48 (34, 79)	0.48
Urine potassium, mEq/L	22 (16, 29)	22 (15, 27)	0.41
Urine glucose, mg/dL	8 (2, 33)	5 (2, 21)	0.14
Urine urea nitrogen, mg/dL	336 (254, 593)	347 (302, 462)	0.77
Urine aquaporin-2, ng/mL	0.95 (0.55, 2.63)	0.66 (0.29, 2.25)	0.73
Urine volume, mL/day	1025 (800, 1320)	1316 (741, 1513)	0.80

Continuous variables are presented as median and interquartile and compared between the two timings by the Wilcoxon signed-rank test. * *p* < 0.05.

**Table 3:** Impact of the potential baseline variables for the change in fractional excretion of sodium after administration of goreisan.

Potential Baseline Variables	Estimated Regression Coefficient	95% CI	*p*-Value	Adjusted *R*-Squared
Univariable analysis				
Age, years	0.038	−0.004–0.080	0.075	0.11
Men	−0.315	−1.060–0.429	0.39	−0.011
Left ventricular ejection fraction, %	−0.004	−0.032–0.025	0.8	−0.049
Equivalent dose of furosemide, mg/day	0.013	−0.005–0.030	0.15	0.057
Urine volume, mL/day	0	−0.001–0.001	0.91	−0.055
Estimated GFR, mL/min/1.73 m^2^	−0.017	−0.042–0.008	0.16	0.06
Serum sodium, mEq/L	−0.026	−0.112–0.060	0.54	−0.031
Logarithm of plasma BNP, pg/mL	0.946	0.207–1.686	0.015 *	0.24
With mineralocorticoid receptor antagonist	−0.497	−1.330–0.336	0.23	0.027
With sodium-glucose cotransporter 2 inhibitor	−0.238	−0.968–0.492	0.5	−0.027
Multivariable analysis				
Model 1				0.29
Age, years	0.029	−0.010–0.068	0.14	
Logarithm of plasma BNP, pg/mL	0.832	0.100–1.565	0.028 *	
Model 2				0.34
Equivalent dose of furosemide, mg/day	0.014	−0.001–0.029	0.06	
Logarithm of plasma BNP, pg/mL	0.999	0.308–1.690	0.0071 *	

Clinically potential baseline variables were included in linear regression analysis. * *p* < 0.05. CI, confidence interval; GFR, glomerular filtration rate; BNP, B-type natriuretic peptide.

## Data Availability

The data presented in this study are available from the corresponding author upon request. The data are not publicly available due to privacy restrictions.
